# Balancing the autonomic scale: the promise of transcutaneous vagus nerve stimulation in combating cardiovascular diseases

**DOI:** 10.3389/fphys.2026.1881568

**Published:** 2026-09-09

**Authors:** Yao Tong, Zhengmeng Ye, Qiuyan Jiang, Chaofu Li, Jun Xiao, Rui Zeng, Hao Wang, Chuanwei Li

**Affiliations:** 1 Department of Cardiology, Chongqing Key Laboratory of Emergency Medicine, Chongqing University Central Hospital (Chongqing Emergency Medical Center), Chongqing University, Chongqing, China; 2 Department of Cardiology, West China Hospital, Sichuan University, Chengdu, China

**Keywords:** arrhythmias, cardiovascular diseases, heart failure, hypertension, transcutaneous vagus nerve stimulation

## Abstract

**Introduction:**

Cardiovascular diseases (CVD) remain a significant burden on global health, often associated with autonomic nervous system (ANS) dysfunction, particularly prolonged sympathetic hyperactivity. Transcutaneous vagus nerve stimulation (tVNS) offers a non-invasive method for modulating vagal tone to achieve autonomic balance, presenting a potentially promising therapeutic approach for hypertension, heart failure and arrhythmia that requires further validation.

**Methods:**

This review elucidates the mechanistic foundations of tVNS and examines its evolving application in the management of cardiovascular diseases. We also critically evaluate current challenges, including the preliminary nature of the evidence, the predominance of surrogate endpoints, and the need for adequately powered multicentre trials to determine whether tVNS can deliver meaningful clinical benefit.

**Results:**

Preliminary evidence suggests that tVNS may modulate heart rate variability, enhance baroreflex sensitivity, and reduce inflammatory markers, although these effects have been demonstrated primarily in small-scale or short-term studies. Available safety data indicate that tVNS is generally well tolerated in the short term, with most reported adverse effects being mild and transient. However, challenges remain in optimizing stimulation parameters, assessing long-term efficacy, and developing personalized treatment protocols.

**Discussion:**

However, it must be emphasized that no major cardiovascular guideline currently recommends tVNS for routine clinical use, and the evidence base remains investigational.

## 1 Introduction

Cardiovascular disease (CVD) remains the leading cause of both disability and mortality worldwide (GBD 2023 Cardiovascular Disease Collaborators, 2025). Evidence has implicated ANS dysregulation in disease development and progression (Mott and Caldwell, 2025). Excessive and sustained sympathetic activation not only accelerates disease progression but may also trigger its early onset (Gronda et al., 2022; He et al., 2024). Contemporary therapeutic approaches predominantly involve pharmacological suppression of sympathetic activity (The Task Force for the management of arterial hypertension of the European Society of Cardiology and the European Society of Hypertension, 2019; McEvoy et al., 2024), however, this strategy is often constrained by patient intolerance or contraindications to these medications (Li et al., 2021). More invasive options, such as surgical sympathectomy, are irreversible and carry significant procedural risks (Dusi et al., 2022). Thus, a fundamental challenge in treatment is the inability to maintain autonomic homeostasis. This deficit contributes to disease progression and elevates the risk of severe cardiovascular complications. The clinical urgency of this issue underscores the necessity for novel strategies aimed at more effectively restoring ANS balance.

While contemporary therapeutic approaches predominantly target the sympathetic nervous system, pharmacological blockade, nerve stimulation, and nerve ablation techniques are progressively being integrated into clinical practice and may represent the next advancement in CVD intervention (Kipshidze et al., 2017; Capilupi et al., 2020; Paton et al., 2026). For instance, ganglion ablation has demonstrated efficacy in reducing the frequency of syncope and obviating the need for permanent pacemaker implantation in cases of refractory vasovagal syncope (Capilupi et al., 2020). There is also a burgeoning interest in treatments that focus on the vagus nerve. Notably, non-invasive neuromodulation, specifically transcutaneous vagus nerve stimulation (tVNS), has shown promising potential in CVD treatment in recent years. Stimulation of the auricular branch of the vagus nerve (ABVN) with tVNS can modulate ANS activity and subsequently influence cardiovascular function (Carandina et al., 2021). Research indicates that tVNS enhances cardiac autonomic activity, as evidenced by increased heart rate variability (HRV) and decreased heart rate in healthy subjects, which holds significant implications for CVD management (Atanackov et al., 2025). Preliminary studies in major CVD models, including heart failure, hypertension and atrial fibrillation (AF), have identified the modulatory effects of this technique on cardiovascular autonomic control mechanisms (Yu et al., 2013; Zhou et al., 2019; Agarwal et al., 2024).

Although there are only a limited number of scattered and inconclusive reports concerning the optimal stimulation settings and effective sham procedures for tVNS, the majority of studies indicate that adverse effects are generally confined to transient sensations of tingling or pain at the site of stimulation (Frangos et al., 2015). Overall, tVNS exhibits a favorable safety and tolerability profile (Mwamburi et al., 2017; von Wrede and Surges, 2021; Gerges et al., 2024). Nevertheless, the further development of tVNS holds promise as a complementary or alternative medical intervention alongside conventional pharmacological and surgical treatments, offering a potential novel approach that warrants further investigation. However, additional large-scale, controlled clinical trials are required to elucidate the mechanisms underlying its efficacy, optimize treatment protocols, and assess the benefits associated with prolonged use.

## 2 Vagal nerve physiology and cardioprotective effects of tVNS

### 2.1 Anatomy and physiological roles of the vagus nerve

**Figure 1:**
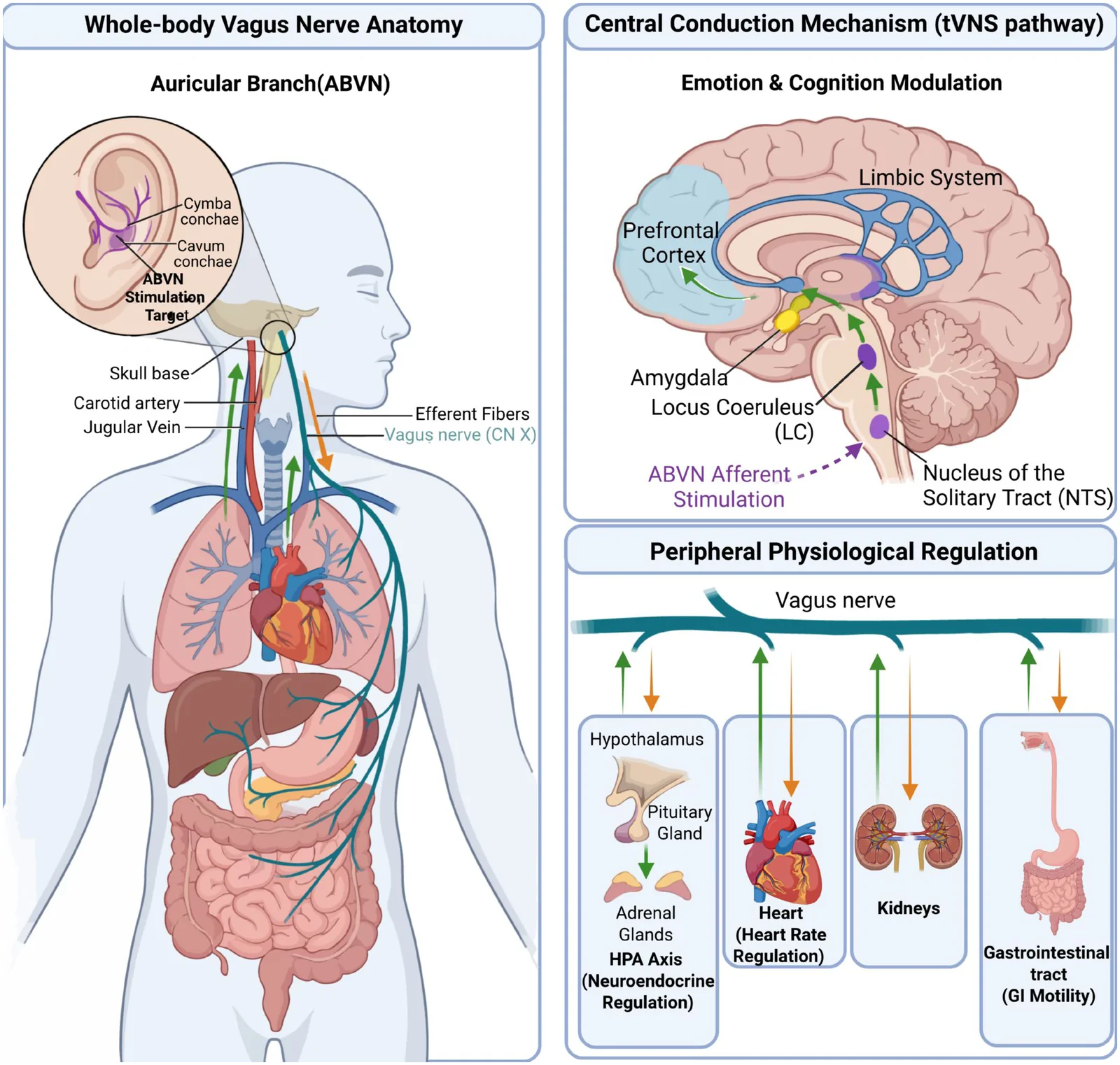
Anatomical target and central pathways of taVNS (transcutaneous auricular vagus nerve stimulation). The auricular branch of the vagus nerve (ABVN) innervates the external auditory canal and concha region, where it is accessible for non-invasive stimulation. Afferent fibers project to the nucleus tractus solitarius (NTS), which relays signals to the locus coeruleus (LC) and amygdala. Solid arrows indicate established anatomical connections. The inset shows typical electrode placement on the cymba concha (active) and tragus/earlobe (sham/control sites). This framework underpins the use of taVNS to modulate autonomic outflow and cardiovascular function.

The parasympathetic nervous system plays a crucial role in maintaining cardiovascular homeostasis, with the vagus nerve being the longest cranial nerve. Its anatomical and physiological functions underpin the theoretical foundation of tVNS (Bonaz et al., 2018). The vagus nerve comprises both afferent and efferent fibers, approximately 80% of which are afferent (Chang et al., 2015). After exiting the skull, the nerve bifurcates into cervical and auricular branches (Hilz, 2022). The ABVN spreads into the external auditory canal and concha area. Its superficial location makes the auricular branch readily accessible for noninvasive stimulation, making it an ideal target (Bermejo et al., 2017). Physiologically, the vagus nerve is vital for maintaining homeostasis, as it regulates functions such as heart rate and gastrointestinal motility, and modulates neuroendocrine reflexes, including the hypothalamic-pituitary-adrenal (HPA) axis (Obata et al., 2020; Gee et al., 2024; Hwang and Oh, 2025; Liu et al., 2025; Xing et al., 2025). Recent research has demonstrated that its afferent fibers have extensive connections through the nucleus tractus solitarius (NTS) to other brain regions, such as the locus coeruleus and the amygdala (Frangos et al., 2015). This provides a neuroanatomical basis for understanding how tVNS may influence emotional and cognitive processing (Frangos et al., 2015; Ma et al., 2025) (**Figure 1**).

### 2.2 Evidence for invasive vagus nerve stimulation and its cardiovascular protective effects

iVNS is an established neuromodulatory therapy approved for refractory epilepsy, treatment-resistant depression and obesity (Badran and Austelle, 2022). Recent studies have also revealed its cardioprotective effects. In a canine model of myocardial ischemia–reperfusion, brief iVNS delivered before reperfusion significantly reduced infarct size and prevented subsequent heart failure; this effect was partly attributable to iVNS-induced bradycardia (Arimura et al., 2017). Additionally, iVNS improves cardiac function by modulating autonomic balance (enhancing vagal tone and suppressing sympathetic activity) and activating the cholinergic anti-inflammatory pathway (Clancy et al., 2014; Arimura et al., 2017). These findings provide important evidence for understanding the cardioprotective mechanisms of non-invasive tVNS.

### 2.3 Modulation of autonomic balance by tVNS

The cardioprotective effects of tVNS are primarily mediated through the modulation of autonomic balance (Toschi et al., 2023; Maestri et al., 2024; Croft et al., 2025; Kaduk et al., 2025). This mechanism operates in two main ways. Firstly, the activation of auricular afferent fibers leads to the inhibition of preganglionic sympathetic neurons, resulting in decreased norepinephrine release (Clancy et al., 2014). Secondly, tVNS enhances parasympathetic efferent signaling, as evidenced by an increase in the high-frequency component of HRV (Gianlorenço et al., 2024; Atanackov et al., 2025; Choudhury et al., 2025). Clinical studies have demonstrated that taVNS can improve baroreflex sensitivity in individuals with heart failure (Gentile et al., 2025) and reduce hypertension in Dahl salt-sensitive rats fed with a high-salt diet (Zhou et al., 2019), indicating its regulatory effects on vasomotor areas through mechanisms like the NTS-hypothalamus axis. Variations in stimulation parameters (e.g., frequency, intensity) and stimulation sites (auricular vs. cervical), and laterality (right vs. left ear) can elicit different autonomic responses, necessitating careful consideration (Keatch et al., 2023; Ludwig et al., 2024; Phillips et al., 2025; Sun et al., 2025). For instance, Atanackov and colleagues investigated the effects of various frequency (10 Hz or 25 Hz) and pulse width (100 µs, 250 µs or 500 µs) combinations on heart rate variability using taVNS. They found that three specific combinations—10 Hz/250 µs, 10 Hz/500 µs and 25 Hz/100 µs—significantly increased SDNN, whereas none of the protocols affected RMSSD, a metric reflecting vagally mediated heart rate variability (Atanackov et al., 2025). Therefore, optimizing the stimulation protocol is crucial for achieving precise neuromodulation.

### 2.4 Anti-inflammatory and antioxidant effects of tVNS

**Figure 2:**
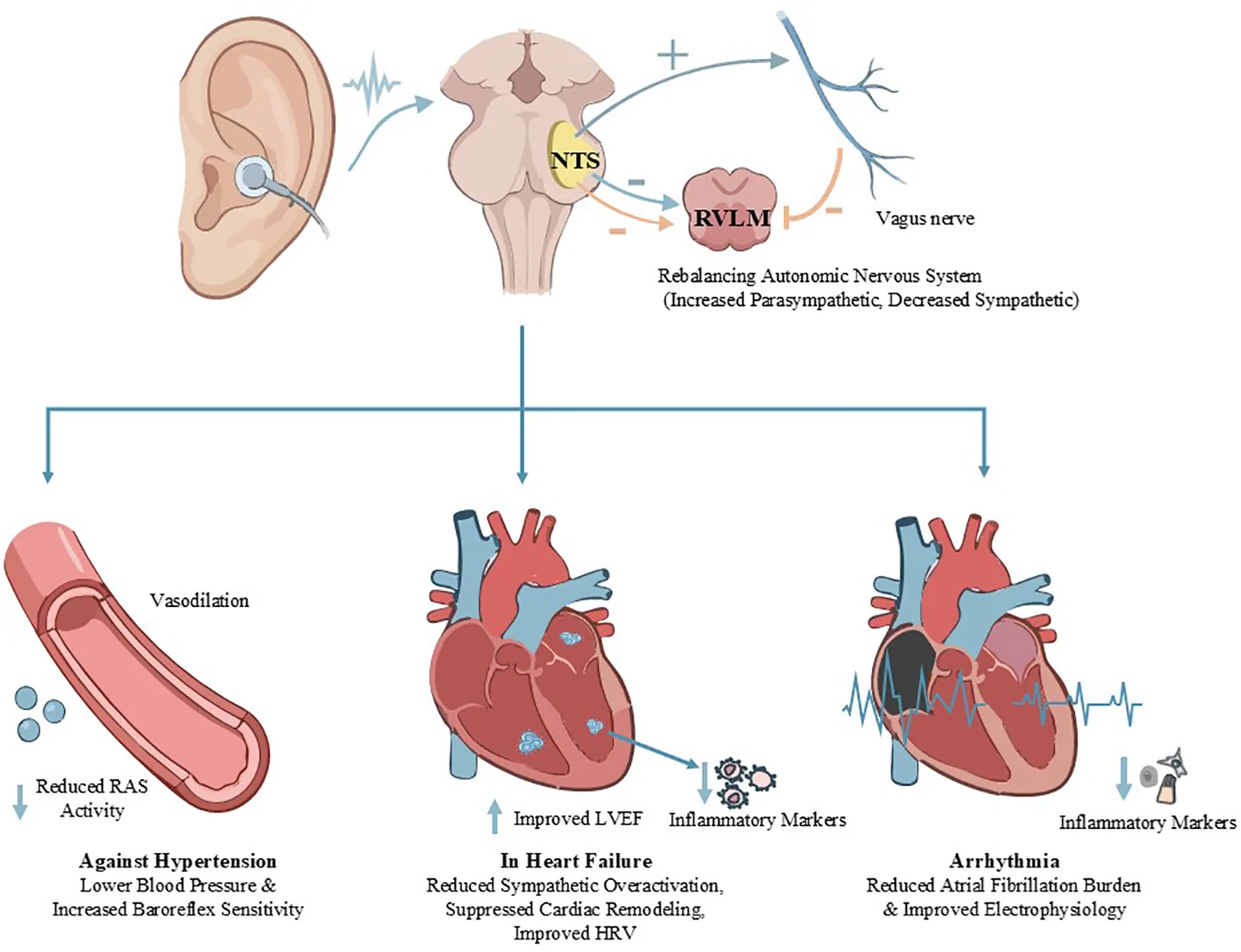
Schematic overview of the proposed mechanisms by which tVNS exerts. Central pathways (NTS→RVLM inhibition, DMNV activation) reduce sympathetic outflow and enhance parasympathetic tone. Peripheral effects include reduced heart rate, increased HRV, improved baroreflex sensitivity, and activation of the cholinergic anti-inflammatory pathway (ACh→α7nAChR→NF-κB inhibition→reduced TNF-α, IL-6). Solid arrows indicate mechanisms supported by direct human evidence; dashed arrows indicate pathways derived predominantly from animal models and remain speculative in CVD.

The vagus nerve modulates immune responses via the cholinergic anti-inflammatory pathway, and tVNS has been shown to engage this same mechanism to reduce inflammation and oxidative stress (Han et al., 2025). Evidence from animal studies suggests that vagus nerve stimulation induces splenic macrophages to release acetylcholine. This neurotransmitter acts on the Alpha-7 nicotinic acetylcholine receptor (α7nAChR) to inhibit the NF-κB pathway, thereby reducing the activity of pro-inflammatory cytokines such as TNF-α and IL-6 (Liu et al., 2025). In human subjects, taVNS has been shown to reduce levels of inflammatory markers, such as C-reactive protein (CRP) (Corrêa et al., 2022), probably through vagus-locus coeruleus-spleen axis activation (Bonaz et al., 2016). Furthermore, tVNS may enhance antioxidant defenses by upregulating the activity of enzymes involved in glutathione synthesis, offering protection against hepatic ischemia-reperfusion injury (Xia et al., 2020). Studies on traumatic brain injury models indicate that tVNS mitigates oxidative stress, inflammation and cell death by inhibiting the NF-κB/NLRP3 pathway ultimately facilitating the recovery of neurological function (Tang et al., 2020). These anti-inflammatory and antioxidant properties are particularly relevant to cardiovascular diseases, where chronic low-grade inflammation and oxidative stress play pivotal roles in disease progression (**Figure 2**).

A distinction must be made between established and speculative mechanisms. Direct human evidence supports sympathoinhibition (Clancy et al., 2014) and HRV enhancement (Atanackov et al., 2025); the anti-inflammatory pathway is validated in humans (non-CVD) and animals (Corrêa et al., 2022). In contrast, antioxidant effects (glutathione synthesis) and NF-κB/NLRP3 inhibition derive mostly from non-CVD animal models (Tang et al., 2020), and their cardiovascular relevance remains speculative.

## 3 Comparison of tVNS with other neuromodulatory approaches

**Figure 3:**
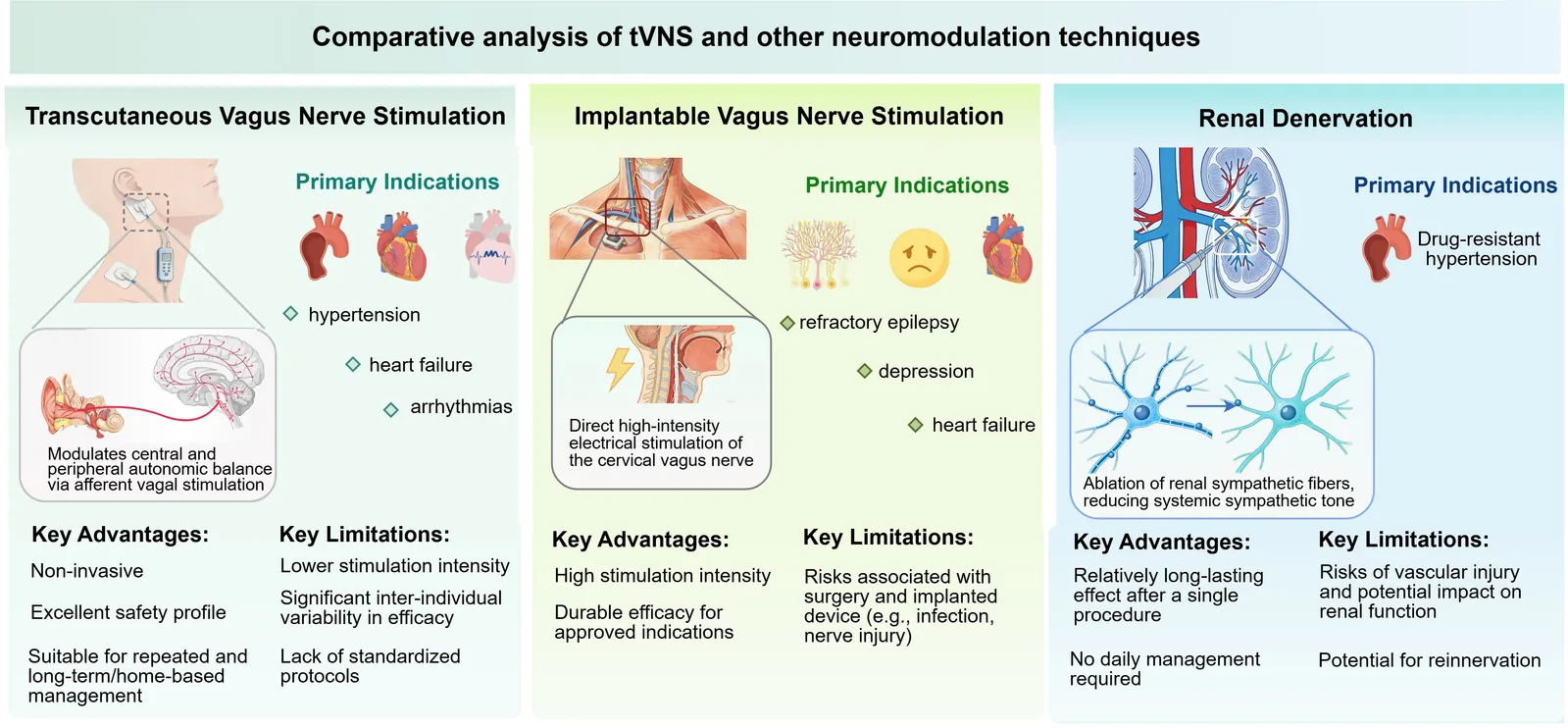
Comparative analysis of tVNS and traditional neuromodulation techniques. The four panels compare invasiveness (tVNS: non-invasive; iVNS/RDN: invasive), stimulation target (ABVN versus cervical vagus versus renal nerves), clinical indications (tVNS: investigational for CVD; iVNS: approved for epilepsy/depression; RDN: approved for resistant hypertension), and safety profile (tVNS: favorable, mild transient effects; iVNS/RDN: procedure-related risks). The comparison highlights tVNS’s unique advantages—non-invasiveness and favorable safety—while acknowledging the need to establish cardiovascular efficacy.

As a non-invasive neuromodulation approach, tVNS differs significantly from traditional methods such as iVNS and renal denervation (RDN) in terms of its underlying mechanisms, clinical indications, safety profile, and applications (**Figure 3**).

### 3.1 Comparison with implantable VNS

iVNS requires surgically implanting electrodes to directly stimulate the cervical vagus nerve, mainly for treating refractory epilepsy and depression (Austelle et al., 2022; Abdennadher et al., 2024), with recent investigations extending to heart failure (Libbus et al., 2022). The first-in-man study of VNS in 32 patients with chronic heart failure demonstrated that VNS was safe and well tolerated, with significant improvements in NYHA class, quality of life, and LVEF at 6 months (De Ferrari and Schwartz, 2011). However, subsequent pivotal randomized controlled trials—NECTAR-HF (Zannad et al., 2015), INOVATE-HF (Gold et al., 2016), and ANTHEM-HFrEF (Konstam et al., 2026)—yielded neutral results for their primary efficacy endpoints, highlighting the complexity of translating VNS from preclinical promise to clinical therapy. In iVNS research, the “neural fulcrum” hypothesis has been proposed as a framework for optimizing stimulation parameters, defined as the operating point—determined by frequency, amplitude, and pulse width—at which opposing afferent and efferent effects on heart rate cancel. This concept may inform tVNS parameter optimization, as the differential responses observed between healthy subjects and heart failure patients to identical tVNS protocols (Maestri et al., 2024) may reflect baseline autonomic status shifting the effective fulcrum position (Ardell et al., 2017). Although iVNS provides high stimulation intensity and precise targeting, it is invasive and carries risks associated with surgery and device-related complications, such as infection and vocal cord paralysis (Wheless et al., 2018; Thompson et al., 2021). Conversely, tVNS stimulates the ABVN via surface electrodes. It is entirely non-invasive, exhibits a higher safety profile, and is generally more acceptable to patients. Nevertheless, its stimulation intensity is lower, and the precision and efficacy of neuromodulation may not be equivalent to that of iVNS (Ellrich, 2019; Jiakai et al., 2023). The iVNS studies in the cardiovascular area have focused on the long-term management of heart failure (Libbus et al., 2022). Given its non-invasive nature, tVNS can be employed earlier, in conjunction with other therapies, and as a long-term management strategy suitable for home application (Hamer and Bauer, 2019).

Although the three major iVNS trials in heart failure (NECTAR-HF, INOVATE-HF, and ANTHEM-HFrEF) all failed to meet their primary endpoints, their failure warrants scrutiny. Possible contributing factors include: suboptimal stimulation parameters that did not achieve effective vagal engagement (Zannad et al., 2015); irreversible autonomic remodeling in advanced heart failure; and the “neural fulcrum” hypothesis, which posits that responses depend on baseline autonomic state (Ardell et al., 2017). Moreover, iVNS delivers mixed afferent and efferent stimulation, whereas tVNS primarily activates afferent fibers, so the therapeutic pathways may differ. These lessons underscore that tVNS optimization requires systematic dose-finding, biomarker-based patient enrichment, and closed-loop strategies—rather than over-optimism based solely on preclinical data.

### 3.2 Comparison with renal denervation

RDN is a catheter-based procedure designed to reduce sympathetic nerve activity by applying radiofrequency or ultrasound energy to ablate the perivascular renal sympathetic fibers (Rao and Krishnan, 2022). There is also experimental evidence that RDN ablates renal afferents, and that part of the therapeutic benefit is achieved through the ablation of these afferents (Evans et al., 2025). It is primarily utilized in the management of resistant hypertension (Camafort et al., 2023; Katsurada and Kario, 2024; Khan and Frishman, 2024; Ogoyama and Kario, 2024). Although RDN offers relatively enduring therapeutic benefits, it is considered a highly invasive procedure with potential adverse effects, including vascular damage and possible impairment of renal function (Ott et al., 2015; Sanders et al., 2017; Dong et al., 2023). Additionally, tVNS has been shown to modulate the central and peripheral balance of the autonomic nervous system, an effect that is also observed with RDN, which ablates both renal efferent and afferent fibers (Clancy et al., 2014). Its therapeutic effects extend beyond blood pressure reduction, potentially enhancing HRV and suppressing inflammation, thereby expanding its applicability to various cardiovascular conditions such as arrhythmias and heart failure (Chen et al., 2020; He et al., 2024). Although both techniques aim to regulate autonomic function, they differ in mechanisms of action, specific clinical indications, and risk profiles. Due to its non-invasive nature, favorable safety profile, and suitability for repeated use, tVNS is theoretically more appropriate for early-stage or mild cardiovascular disease intervention, or as a part of combination therapy strategies (He et al., 2024).

## 4 Clinical benefits, safety profile, and applicability of tVNS across different CVD

**Figure 4:**
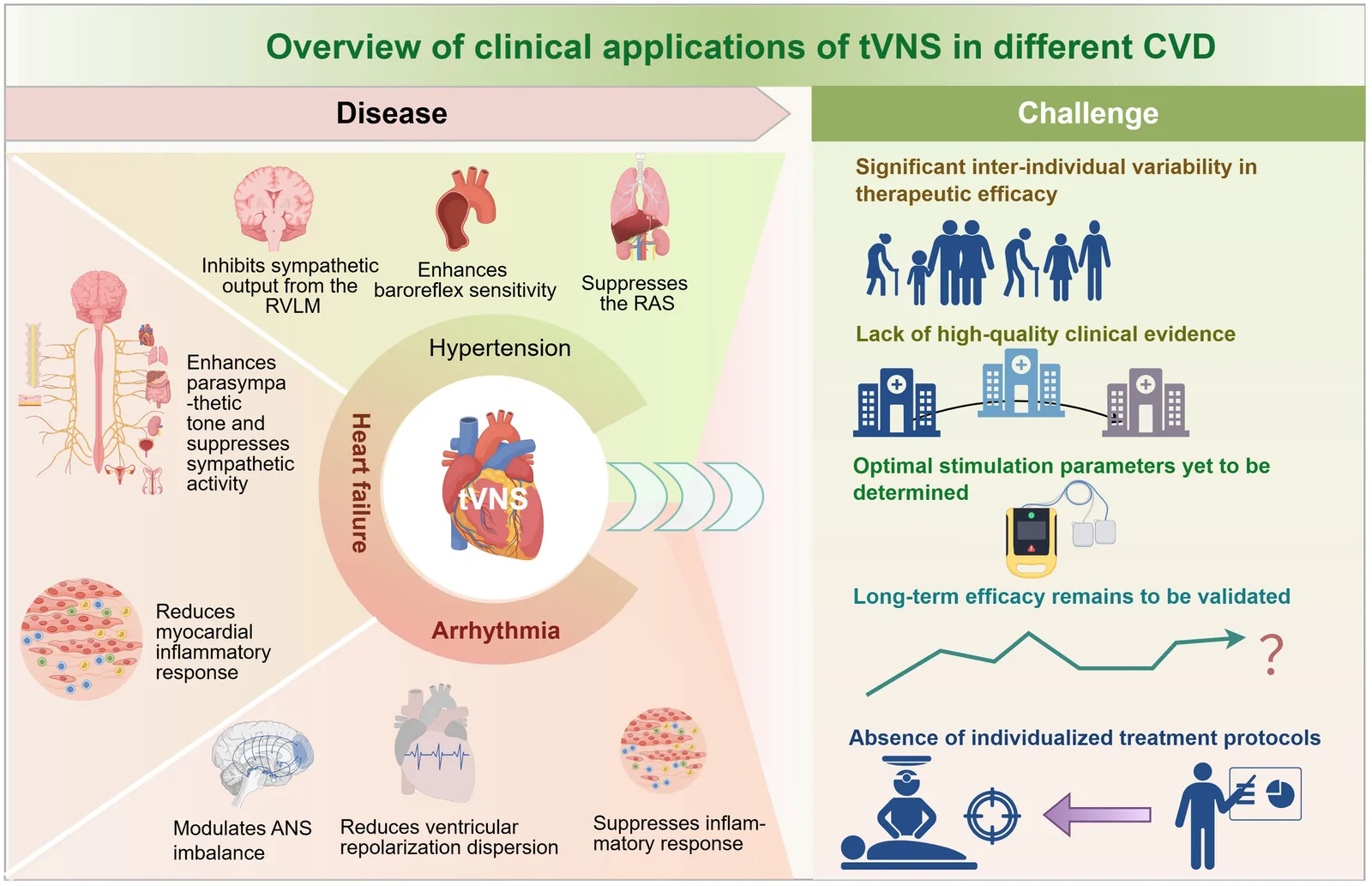
Overview of clinical applications of tVNS in different CVD. For each condition, the figure shows proposed mechanisms, key clinical studies, and reported effects. An evidence-level bar indicates the strength of evidence: moderate for surrogate endpoints in heart failure (based on meta-analyses), low to very low for hard clinical outcomes across all conditions, and no demonstrated mortality or hospitalization benefit. Solid lines indicate mechanisms with direct human evidence in cardiovascular populations; dashed lines indicate pathways requiring further clinical confirmation. tVNS remains investigational and is not recommended by any major cardiovascular guideline for routine clinical use.

Based on the aforementioned mechanisms of autonomic regulation and anti-inflammatory action, tVNS has demonstrated therapeutic potential across a spectrum of cardiovascular conditions. This section reviews the available evidence for its application in hypertension, heart failure, and arrhythmias, followed by a comprehensive assessment of its safety profile (**Figure 4**; **Table 1**).

**Table 1:** Stimulation parameters and key clinical responses of tVNS in different cardiovascular diseases.

Condition	Sample size	Stimulation site	Frequency (Hz)	Intensity	Protocol	Key clinical/physiological responses	Study (Reference)
Healthy adults	NR	taVNS (external acoustic meatus)	300	2–8.1 mA (below perceptual threshold)	Single session; 30 s	2–4.8 mA increased pupil size; higher intensities (up to 8.1 mA) did not	Phillips et al.; 2025 (Phillips et al., 2025)
Healthy adults	29	taVNS (left tragus)	10; 25	Above perceptual threshold	Single session	Left 25 Hz more effective than 10 Hz in reducing HR (-1.0 vs -0.5 bpm)	Maestri et al.; 2024 (Study 1) (Maestri et al., 2024)
Healthy adults	28	taVNS (left vs right tragus)	25	Above perceptual threshold	Single session	Left > right for HR reduction (-0.9 vs -0.3 bpm)	Maestri et al.; 2024 (Study 2) (Maestri et al., 2024)
Healthy adults	30	taVNS (left tragus)	25	Above perceptual threshold	Single session	Left 25 Hz significantly reduced HR (-1.1 ± 1.2 bpm; p < 0.01); sham no effect	Maestri et al.; 2024 (Study 3) (Maestri et al., 2024)
Hypertension	20	taVNS (cymba concha)	2; 10; 25; 100	Sub- to supra-perceptual threshold	RAVANS; five sessions	100 Hz significantly reduced diastolic BP and mean arterial pressure	Garcia et al.; 2022 (Garcia et al., 2022)
Heart failure (stable CHF)	13 (12 completed)	taVNS (triangular fossa)	1	1 mA	40 min stimulation + 20 min break	6-MWD increased by 101.1 m (p = 0.04); no change in CRP or NT-proBNP; no adverse events	Schukro et al.; 2025 (Schukro et al., 2025)
Heart failure (CHF; LVEF < 50%)	16	tcVNS (transcutaneous cervical vagus nerve stimulation) (right vs left tragus)	30	1 mA below discomfort threshold	Two 10-min trials; randomized order	Right-sided tVNS increased cardio-vagal baroreflex gain (5.6 → 7.5 ms/mmHg; Δ1.9; p < 0.001); left-sided no effect; no adverse effects	Gentile et al.; 2024 (Gentile et al., 2025)
Heart failure (HFrEF)	43	taVNS	2/15	Low-intensity	Prospective; double-blind; sham-controlled	Improved rMSSD (31 vs 21; p = 0.046) and SDNN (110 vs 84; p = 0.033)	Couceiro et al.; 2023 (Couceiro et al., 2023)
Heart failure (HF)	32	taVNS (left tragus)	25	Above perceptual threshold	Single session	Active and sham both produced negligible effects (unlike healthy subjects)	Maestri et al.; 2024 (HF patients) (Maestri et al., 2024)
Heart failure (acute)	22	LLTS (low-level tragus stimulation) (left tragus vs earlobe)	20	1 mA	1 h/day × 5 days	2023: Reduced CASP, SBP, DBP, and HR (all p < 0.05); acute afterload reduction. 2024: Reduced SBP variability (SD, CV, δ) (all p < 0.05)	Nagai et al.; 2023 & 2024 (Nagai et al., 2023; Nagai et al., 2024)
Atrial fibrillation	26 active; 27 sham	LLTS (tragus)	20	1 mA below discomfort threshold	1 h/day; 6 months	Reduced AF burden (median reduction 85%; OR 0.15); suppressed inflammation	Stavrakis et al.; 2020 (TREAT AF) (Stavrakis et al., 2020)
Atrial fibrillation	120 (planned)	tVNS	NR	NR	Daily; 6-month follow-up	Ongoing RCT; primary endpoint: AF recurrence rate	Swojanowsky et al.; 2024 (VAST-AF) (protocol) (Swojanowsky et al., 2024)
PVCs	36 (35 completed)	LLTS (tragus)	20	1 mA below discomfort threshold	Two 10-day sessions; 8-day washout	PVC burden reduced by 13.4% (10.5% vs 8.59%; p = 0.021); more pronounced in slow HR-dependent PVCs	Zafeiropoulos et al.; 2025 (NoVa-PVC) (Zafeiropoulos et al., 2025)
PVCs	Planned	taVNS	NR	NR	6-week treatment; 12-week follow-up	Ongoing RCT; primary outcome: ≥50% PVC reduction on 24-h Holter	Liu et al., 2024 (TASC-V trial protocol) (Liu et al., 2024)
STEMI	110 (55 active; 55 sham)	tVNS (tragus vs earlobe sham)	NR	NR	On admission; throughout PCI; +30 min	Reduced hospital mortality (p = 0.024); cardiogenic shock (p = 0.044); AV block 3° (p = 0.013); composite MACE p = 0.0016	Kruchinova et al.; 2025 (Kruchinova et al., 2025)
Subarachnoid hemorrhage	24	taVNS	NR	NR	ICU stay	No bradycardia or QT prolongation; repetitive taVNS increased overall HRV and parasympathetic activity (Cohen’s d = 0.50); acutely increased HR; BP; and peripheral perfusion index without affecting QTc or ICP	Tan et al.; 2025 (Carandina et al., 2021)
POTS	57	LLTS (tragus)	NR	NR	Controlled; prospective; randomized	No increased risk of local skin irritation or systemic AEs; safe and well-tolerated	Stavrakis et al.; 2024 (Stavrakis et al., 2024)

### 4.1 Application of tVNS in hypertension

tVNS has been investigated for its potential effects on blood pressure regulation, though the evidence remains preliminary. This approach indirectly influences blood pressure by modulating autonomic balance. Empirical studies have demonstrated that taVNS can enhance the high-frequency component of HRV, thereby augmenting parasympathetic tone and subsequently reducing both heart rate and blood pressure (Capilupi et al., 2020; Atanackov et al., 2025). The antihypertensive mechanism of tVNS involves a multi-step, multi-channel synergistic process. First, tVNS stimulates vagal afferent fibers. This leads to inhibition of sympathetic outflow from the rostral ventrolateral medulla. The net effect is decreased peripheral vascular resistance and reduced cardiac workload (Kruchinova et al., 2025). Additionally, taVNS may enhance baroreflex sensitivity, thereby improving short-term blood pressure regulation (Kruchinova et al., 2025).

If confirmed in larger trials, tVNS might offer a non-pharmacological adjunctive option. However, no adequately powered randomized controlled trial has yet demonstrated that tVNS provides sustained blood pressure reduction superior to sham or comparable to standard antihypertensive therapy (Paket, 2025). Consequently, it is particularly suitable as an adjunctive or supplementary therapy for patients with resistant hypertension or those who exhibit drug intolerance (Giollo-Junior et al., 2023). Nonetheless, the application of tVNS in managing hypertension encounters several challenges. Firstly, its efficacy is highly variable among individuals and often necessitates prolonged, long-term treatment. Secondly, there is a lack of large-scale RCTs directly comparing tVNS with standard antihypertensive treatments. A review of vagus nerve stimulation therapies noted that while iVNS is an established therapy for epilepsy and depression, data from large-scale, long-term clinical trials evaluating its direct antihypertensive effects are still needed (Carandina et al., 2021). Furthermore, a review on transcutaneous VNS highlighted that its potential application to conditions like hypertension and atrial fibrillation is promising but primarily based on mechanistic and pre-clinical evidence, underscoring the need for more dedicated clinical research (Arimura et al., 2017). Future research should address these issues by focusing on optimizing stimulation parameters, identifying patient populations most likely to benefit, and establishing its definitive role as a treatment through rigorously designed clinical trials (Garcia et al., 2022).

### 4.2 Application of tVNS in heart failure

Heart failure is characterized by a significant autonomic imbalance, marked by heightened sympathetic activity and diminished parasympathetic activity, which contribute to increased cardiac remodeling and functional decline (Wu et al., 2025). tVNS has been proposed as a potential neuromodulatory approach to restore autonomic balance in heart failure, but this remains investigational (Jiang et al., 2025). The primary mechanism by which tVNS exerts its cardioprotective effects involves the enhancement of parasympathetic tone and the attenuation of sympathetic activity. This modulation immediately results in decreased heart rate, enhanced HRV, and suppression of myocardial inflammation (Geng et al., 2022; Gianlorenço et al., 2024; Maestri et al., 2024; Sun et al., 2025). Preclinical studies and small clinical trials have reported improvements in surrogate measures such as LVEF and 6-minute walking distance, but these findings require confirmation in larger, longer-term studies with hard clinical endpoints (Zhou et al., 2019). However, the precise molecular mechanisms underlying these effects remain to be elucidated. A clinical study found that acute right-sided transcutaneous vagus nerve stimulation improved cardio-vagal baroreflex gain in patients with chronic heart failure (Gentile et al., 2025), providing direct evidence for its potential to restore autonomic balance in this population.

At present, the long-term efficacy data for tVNS in treating heart failure remain limited. Support for the cardioprotective concept of VNS comes from animal studies; for instance, intravenous VNS in a canine model of myocardial infarction significantly reduced infarct size and preserved cardiac function, an effect partly attributed to its ability to modulate autonomic balance and induce bradycardia (Arimura et al., 2017). Similarly, intraoperative taVNS accelerated postoperative recovery and improved gastric function in a rat model by enhancing vagal tone and suppressing inflammatory cytokines (Elkholey et al., 2022), a mechanistic pathway relevant to heart failure pathology. However, large-scale randomized controlled trials of invasive VNS (iVNS) in HFrEF have yielded conflicting results. The INOVATE-HF trial showed that iVNS did not reduce the risk of death or heart failure events (Gold et al., 2016); the NECTAR-HF trial failed to demonstrate improvement in left ventricular remodeling (Zannad et al., 2015); and the ANTHEM-HFrEF trial reported a neutral primary endpoint (Konstam et al., 2026). These discrepancies underscore the complexity of translating VNS from preclinical studies to clinical therapy and highlight the urgent need for rigorously designed trials to validate tVNS protocols.

Future studies with extended follow-up are essential to evaluate not only its effects on critical endpoints such as rehospitalization and mortality rates but also to monitor potential neural adaptive changes resulting from chronic stimulation. Consequently, it is imperative to conduct prospective, multicenter clinical trials with prolonged follow-up to conclusively determine the long-term efficacy and safety profile of tVNS.

### 4.3 Application of tVNS in arrhythmia

The therapeutic efficacy of tVNS for arrhythmias exhibits considerable variability, with outcomes strongly influenced by the specific type of arrhythmia being treated and the parameters of stimulation employed. Small studies have reported that low-level taVNS may reduce heart rate and AF burden in some patients with paroxysmal AF, though these findings are preliminary., while maintaining a favorable safety profile and alleviating symptoms (Jiang et al., 2025). Furthermore, chronic, intermittent low-level taVNS has been shown to decrease AF burden and simultaneously enhance HRV and inflammatory markers (Stavrakis et al., 2020). However, the effects of vagus nerve stimulation on AF are complex and bidirectional, capable of producing either pro-arrhythmic or anti-arrhythmic outcomes, depending primarily on the specific stimulation site and parameters (Salavatian et al., 2016). Consequently, identifying the optimal stimulation regimen and elucidating the underlying mechanisms represent critical avenues for future research.

In the field of ventricular arrhythmias, tVNS primarily functions to prevent the onset of lethal arrhythmias through its electrophysiological effects, such as reducing the dispersion of ventricular repolarization and inhibiting early afterdepolarizations (Jiang et al., 2020). Its clinical efficacy has been preliminarily validated in patients with acute myocardial infarction; a RCT demonstrated that taVNS significantly decreased in-hospital mortality, the incidence of cardiogenic shock, and third-degree atrioventricular block, while also attenuating myocardial injury and reducing the occurrence of severe arrhythmias (Kruchinova et al., 2025). Additionally, tVNS influences inflammatory markers, including C-reactive protein, interleukin-10, and interferon-γ, which may indirectly enhance arrhythmia prognosis (Stavrakis et al., 2015). A recent perspective article highlights the potential of transcutaneous vagus nerve stimulation as a novel neuromodulatory therapy for atrial fibrillation and ischemic stroke (Förster, 2024). This is supported by evidence that cervical non-invasive VNS can activate vagal afferent fibers, as measured by vagal somatosensory evoked potentials (Nonis et al., 2017), and that taVNS paired with tones modulates auditory cortex oscillations (Rufener et al., 2023), indicating a capacity to influence neural networks relevant to cardiac electrophysiology. With further validation through adequately powered trials, tVNS may eventually find a role as an adjunctive therapy in selected arrhythmia patients.

### 4.4 Safety profile of tVNS

Numerous studies have affirmed the safety of tVNS, a non-invasive neuromodulation technique (Giraudier et al., 2025). Adverse effects associated with tVNS are predominantly mild and transient, as evidenced in various patient cohorts, including individuals with CVD. The most frequently reported adverse effects are localized skin reactions at the stimulation site (erythema or pruritus), headache and dizziness (Giraudier et al., 2025). These symptoms typically resolve spontaneously without the need for intervention (Bauer et al., 2016; Redgrave et al., 2018; Deligiannidis et al., 2022; Kim et al., 2022; Wu et al., 2024; Giraudier et al., 2025). Particularly noteworthy is the safety profile recorded in CVD patient groups. Clinical trials involving patients with conditions such as acute myocardial infarction or heart failure have demonstrated that tVNS does not induce clinically significant bradycardia, QT interval prolongation, or serious hemodynamic disturbances (Kruchinova et al., 2025). Although a single acute session of tVNS may cause a minor, transient increase in heart rate or blood pressure, this response has been shown to diminish with repeated exposure to stimulation (Kruchinova et al., 2025). Of note, this observation was derived from patients with subarachnoid hemorrhage, in whom repetitive taVNS during intensive care unit stay did not cause bradycardia or QT prolongation and was associated with increased overall heart rate variability and parasympathetic activity (Tan et al., 2025). These findings underscore the excellent safety profile of tVNS when utilized in cardiovascular applications.

The literature across other diseases also confirms and expands on these safety findings. There was no increased risk of local skin irritation during taVNS in patients with postural tachycardia syndrome (POTS), and taVNS was not associated with increased risk of systemic adverse effects (e.g., dizziness, headache) or severe adverse events (Stavrakis et al., 2024). Research involving patients with tinnitus and epilepsy indicates that the side effects of taVNS, such as ear pain, are predominantly mild to moderate in severity. These side effects can typically be managed effectively by adjusting stimulation parameters, with the exception of severe complications, such as a pronounced vasovagal response, which are exceedingly rare (Lin et al., 2024; Moro et al., 2025). While the adverse effect profile of tVNS is largely consistent across conditions, individual sensitivity varies; therefore, a valuable strategy to enhance safety and tolerability is to assess baseline vagal tone (e.g., via respiratory sinus arrhythmia) prior to treatment initiation, allowing for personalized optimization of stimulation parameters (Kovacic et al., 2020).

## 5 Current challenges and limitations of tVNS

Despite the promising therapeutic potential of tVNS, several significant challenges and limitations must be addressed before its widespread clinical adoption. These challenges span technical, methodological, and clinical domains.

### 5.1 Parameter heterogeneity and standardization

The effectiveness of tVNS largely hinges on optimizing stimulation parameters, such as intensity, frequency, and pulse width, which are crucial determinants of its effects (Keatch et al., 2023; Owens et al., 2024; Rezapour et al., 2024). However, existing studies reveal significant variability in these parameters, and no standardized guidelines have been established. Different parameter combinations often produce distinct physiological responses, a characteristic that supports precision neuromodulation but complicates cross-study comparisons.

The stratification of stimulation intensity is often overlooked. While most taVNS studies use intensities above the perceptual threshold to adequately activate afferent fibers (Lench et al., 2023), even subtle variations within this high-intensity range can significantly alter neural response patterns (Owens et al., 2024). Phillips and colleagues reported a non-linear relationship between 300 Hz taVNS intensity and pupil diameter: intensities of 2–4.8 mA increased pupil size, whereas higher intensities (up to 8.1 mA) did not produce this effect (Phillips et al., 2025). Ludwig et al. compared the effects of 3 mA and 5 mA on pupillary dilation, finding that the higher intensity (5 mA) caused more pronounced dilation, suggesting intensity might have a stronger influence than frequency (Ludwig et al., 2024). Animal studies offer complementary mechanistic insights: in α-chloralose-anaesthetized Sprague–Dawley rats, increasing taVNS intensity augments both the number of responsive neurons in the spinal trigeminal nucleus and the response magnitude of individual neurons (Owens et al., 2024). This evidence collectively suggests an optimal high-intensity window exists, beyond which effects may diminish.

Frequency significantly affects the specificity of neuromodulation. fMRI studies reveal that 100 Hz taVNS primarily activates brainstem nuclei like the NTS and locus coeruleus, while activation patterns vary significantly with 2 Hz, 10 Hz, and 25 Hz stimulation (Sclocco et al., 2020). This indicates that frequency selection can determine which anatomical targets are engaged. The clinical relevance of these parameter-effect relationships is demonstrated in disease-specific research. For instance, the 20 Hz/200 µs combination is frequently used to enhance heart rate variability in heart failure patients and has been applied in studies of acute myocardial infarction (Owens et al., 2024). Additionally, research on respiratory-gated taVNS (RAVANS) shows that different stimulation frequencies have varying effects on heart rate and blood pressure (Garcia et al., 2022). These findings suggest that optimal parameters should be customized to specific disease states and therapeutic objectives. The heterogeneity of stimulation parameters across published studies is striking. Stimulation sites range from the tragus and cymba concha to the cervical region; frequencies span from 1 Hz to 300 Hz without clear dose-finding justification (Sclocco et al., 2020); pulse widths vary from 100 µs to 500 µs; intensity definitions (perceptual threshold versus fixed mA) and treatment durations (single session versus months) differ substantially. This marked variability, combined with inconsistent sham protocols, severely hampers cross-study comparisons, obscures the identification of optimal regimens for specific indications, and underscores the urgent need for factorial-design dose-finding studies.

### 5.2 Limitations of current evidence

Most studies to date have been exploratory in nature, characterized by small sample sizes, short follow-up periods, and diverse stimulation conditions. These factors have resulted in considerable variability in reported efficacy, complicating the ability to conduct reliable meta-analyses and draw definitive conclusions. Furthermore, many studies lack rigorous sham-controlled designs. For instance, while one study reported a significant impact of tVNS on in-hospital mortality rates among patients with acute myocardial infarction, it did not demonstrate a statistically significant difference in overall survival at 12 months (Kruchinova et al., 2025). This observation underscores the necessity for more comprehensive research, including extended follow-up periods, to validate these outcomes.

### 5.3 Technical limitations

The existing tVNS technology encounters challenges in standardizing parameters, as critical variables such as stimulation frequency, pulse width, and intensity necessitate dynamic adjustments based on the specific disease context and individual patient response (Thompson et al., 2021; Wang et al., 2022; Naparstek et al., 2023). For example, research involving patients with Prader-Willi syndrome revealed that a daily 4-hour stimulation regimen over nine months markedly reduced emotional outbursts, whereas decreasing the duration to two hours reduced the treatment’s efficacy, thereby illustrating a clear correlation between treatment duration and therapeutic outcomes (Manning et al., 2019; Schmausser et al., 2024). The technical limitations of current devices, including battery life, electrode placement accuracy, and the lack of real-time feedback mechanisms, further constrain the optimization and personalization of tVNS therapy.

### 5.4 The challenge of sham stimulation and placebo effects

Sham control is a major methodological challenge in tVNS trials. Earlobe stimulation, a common sham, is not nerve-free (it is innervated by the great auricular nerve) and its different sensation may unblind participants (Stavrakis et al., 2020). Minimal-intensity stimulation preserves blinding but may deliver subliminal physiological effects; alternative auricular sites assume no therapeutic effect, but this lacks proof. Inadequate sham controls inflate placebo responses and confound specific neuromodulation with non-specific effects. We recommend multi-site sham designs that balance sensation, formal blinding assessment (e.g., Bang Blinding Index), and objective endpoints to improve evidence reliability.

To avoid redundant enumeration, the recurring themes across this review—autonomic imbalance, HRV improvement, sympathoinhibition, inflammation reduction, parameter optimization, and the need for multicenter trials—reflect the central unresolved questions in the field. Rather than reiterating each individually, we emphasize that these interconnected challenges collectively underscore the gap between mechanistic promise and clinical proof.

### 5.5 Regulatory barriers

Currently, no transcutaneous vagus nerve stimulation (tVNS) device has received FDA approval or CE marking for any cardiovascular indication. Although several auricular and cervical tVNS devices have obtained regulatory clearance for neurological or pain-related conditions (e.g., epilepsy, migraine, and opioid withdrawal), their use in hypertension, heart failure, or arrhythmia remains investigational and outside approved labeling (Ingram et al., 2026). This regulatory gap reflects the preliminary nature of the evidence base, which is characterized by small sample sizes, surrogate endpoints, and heterogeneous stimulation protocols (Thompson et al., 2021). Until adequately powered, sham-controlled trials demonstrate clinically meaningful benefits on hard cardiovascular outcomes, tVNS cannot be recommended for routine clinical use in cardiology (Gerges et al., 2024). Future pivotal studies should align with regulatory expectations to support potential marketing authorization (Ingram et al., 2026).

## 6 Quality of evidence and methodological limitations

A critical appraisal of the current literature indicates that the evidence base for tVNS in cardiovascular diseases remains preliminary, and several methodological limitations constrain the strength of any therapeutic claims.

Sample sizes across published studies are consistently small. Most clinical investigations include fewer than 30 patients per treatment arm, and many are pilot or feasibility studies rather than adequately powered confirmatory trials. For instance, the pilot study by Schukro and colleagues enrolled only 12 patients who completed the protocol (Schukro et al., 2025). Likewise, the NoVa-PVC trial, a crossover randomized study, included 36 participants (Zafeiropoulos et al., 2025). These investigations offer valuable preliminary data and help inform future research directions, but they lack the statistical power to detect clinically meaningful differences in hard cardiovascular endpoints.

Another major concern is the predominance of surrogate markers as primary outcomes. Heart rate variability indices, inflammatory biomarkers (including C-reactive protein, TNF-α, and interleukin-6), and echocardiographic parameters are frequently used as surrogate endpoints. While mechanistically informative and biologically plausible, these surrogates do not directly capture outcomes of greatest relevance to patients—such as mortality, hospitalization, quality of life, or major adverse cardiovascular events. A recent systematic review and meta-analysis of ten RCTs comprising 374 heart failure patients reported that tVNS significantly increased LVEF and 6-minute walking distance while reducing TNF-α levels (Sun et al., 2025). However, the clinical relevance of a 3.21% absolute improvement in LVEF remains uncertain, and none of the included trials demonstrated a mortality benefit.

The considerable heterogeneity in stimulation parameters across studies further limits comparability and reproducibility. Reported frequencies range from 1 Hz to 300 Hz, intensities vary from sub-perceptual to supra-perceptual thresholds, stimulation sites differ (auricular tragus versus cymba concha versus cervical), and treatment durations extend from single sessions to six months (Ludwig et al., 2024; Phillips et al., 2025). This variability, combined with inconsistent sham protocols (Gerges et al., 2024), makes it difficult to determine which specific stimulation regimens are most effective for which patient populations. One systematic review of taVNS effects on heart rate variability and baroreflex sensitivity in healthy subjects concluded that the findings were mixed, largely attributable to heterogeneity in study designs and stimulation delivery protocols (Soltani et al., 2023).

When assessed with standardized tools, the overall quality of evidence is low. A recent systematic review applying GRADE methodology rated all efficacy outcomes for taVNS in clinical populations as having “very low” certainty, with safety rated as “low” certainty (Giraudier et al., 2025). This reflects the predominance of small, single-center, unblinded or inadequately blinded studies, together with the frequent absence of pre-registered analysis plans.

Finally, the translation of promising preclinical findings into tangible clinical benefit has proven difficult. The neutral results of major invasive VNS trials in heart failure—NECTAR-HF (Zannad et al., 2015), INOVATE-HF (Gold et al., 2016), and ANTHEM-HFrEF —highlight the gap between mechanistic rationale and clinical efficacy. Although tVNS offers clear advantages over implantable devices in terms of safety and accessibility, it cannot be assumed that the same challenges encountered in iVNS trials will not also apply to tVNS.

Based on the methodological limitations discussed above, we propose the following evidence-level classification for tVNS in cardiovascular applications: for heart failure, the evidence is moderate for surrogate endpoints (LVEF, 6-MWD, inflammatory markers) based on meta-analyses of small RCTs (Sun et al., 2025), but very low for hard clinical outcomes (mortality, hospitalization) (Zannad et al., 2015; Gold et al., 2016; Konstam et al., 2026). For hypertension and arrhythmias, the evidence is low to very low, with few sham-controlled trials (Stavrakis et al., 2020; Swojanowsky et al., 2024) and none demonstrating sustained benefit on clinically meaningful endpoints. This classification underscores that tVNS, while mechanistically promising, remains an investigational therapy requiring further validation.

## 7 Future directions and conclusions

### 7.1 Technical refinement and personalized treatment

Future research should prioritize the development of biomarker-based personalized protocols to quantitatively assess the effects of neuromodulation (Tu et al., 2018). The integration of neuroimaging, dynamic physiological monitoring, and biomarkers will be crucial for personalizing stimulation protocols and identifying individuals who are most likely to respond favorably. Defining and standardizing these parameter-response characteristics is crucial for advancing tVNS toward clinical application. Optimizing individualized stimulation parameters to achieve precise, high-intensity neuromodulation is essential for realizing precision therapy. Future research should incorporate neurophysiological biomarkers like gamma oscillations, evoked potentials, and pupillary responses to develop parameter-effect prediction models. This approach would facilitate the transition of tVNS from its largely empirical use to more precise and individualized neuromodulation.

Moreover, the use of non-auricular points of stimulation (e.g., cervical) can also expand its therapeutic applications (Ottaviani et al., 2022; Wang et al., 2024; Gentile et al., 2025). The advancement of closed-loop stimulation systems, which are capable of real-time feedback adjustment, holds promise for significantly enhancing treatment precision (Yu et al., 2021). Such systems could automatically adjust stimulation parameters based on physiological responses, thereby optimizing therapeutic efficacy while minimizing adverse effects.

### 7.2 Prospective outlook for multicenter clinical trials

In order to overcome current shortcomings, future studies should focus on multicenter randomized controlled trials (RCTs) using large samples and long-term follow-up and standardized efficacy endpoints. These considerations collectively underscore the necessity for more comprehensive research, including extended follow-up periods, to validate these outcomes. Simultaneously, examining various disease states may provide valuable insights into the common mechanisms underlying vagal nerve modulation, potentially establishing a foundation for the development of combination therapies (Veldman et al., 2025). In the long term, standardized devices and operational protocols will be essential for the clinical application of tVNS.

### 7.3 Integration into clinical practice

Based on its mechanism and technical profile, tVNS holds unique potential across the continuum of cardiovascular care. In the realm of primary prevention, it functions as a supplementary approach to lifestyle interventions, facilitating early modulation. During the stable phase of treatment, tVNS contributes to rehabilitation efforts aimed at enhancing prognosis. In cases of refractory or drug-intolerant conditions, it emerges as a crucial element of combination therapy. The successful integration of tVNS into clinical pathways is contingent upon rigorous, large-scale, multicenter prospective trials that validate its long-term safety and efficacy. Establishing an evidence-based, standardized framework is imperative.

## 8 Conclusions

tVNS represents an emerging area of investigation in cardiovascular medicine, offering a non-invasive approach to autonomic modulation that may eventually complement existing therapies. The fundamental mechanism of tVNS involves the modulation of autonomic imbalance through external stimulation, specifically by augmenting parasympathetic activity and attenuating sympathetic overactivity. This therapeutic approach addresses the neural origins of pathological processes in conditions such as hypertension, heart failure, and arrhythmias (Wang et al., 2019; Wang et al., 2024; Talishinsky et al., 2025), thereby providing a complementary clinical option for patients who exhibit suboptimal responses or have contraindications to conventional pharmacological treatments.

Recent research highlights a fundamental paradox: while preliminary studies demonstrate promising efficacy, there remains an absence of standardized clinical protocols. The inconsistency and lack of reproducibility in outcomes can be attributed to significant variations in stimulation targets (such as auricular versus cervical), parameters (including frequency, intensity, and waveform), and treatment durations across different studies (Thompson et al., 2021; Maestri et al., 2024; Baig et al., 2025). Consequently, future research should transition from merely establishing efficacy to delineating optimal therapeutic strategies. The path forward requires a concerted effort to standardize stimulation protocols, validate long-term safety and efficacy through large-scale multicenter trials, and develop personalized approaches based on individual patient characteristics and biomarkers. Addressing these challenges through rigorously designed, adequately powered, sham-controlled trials with long-term follow-up and patient-important outcomes will be essential to determine whether tVNS can transition from a promising experimental intervention to an evidence-based component of cardiovascular care.

## Data Availability

The original contributions presented in the study are included in the article/supplementary material. Further inquiries can be directed to the corresponding authors.
